# Ionizing Radiation Perturbs Cell Cycle Progression of Neural Precursors in the Subventricular Zone Without Affecting Their Long-Term Self-Renewal

**DOI:** 10.1177/1759091415578026

**Published:** 2015-03-27

**Authors:** Hongxin Chen, Matthew T Goodus, Sonia M de Toledo, Edouard I Azzam, Steven W Levison, Nizar Souayah

**Affiliations:** 1 Department of Neurology and Neurosciences, Rutgers University—New Jersey Medical School, Newark, NJ, USA; 2 Department of Radiology, Rutgers University—New Jersey Medical School, Newark, NJ, USA

**Keywords:** cell death, radiation-induced cell cycle checkpoints, brain, cell differentiation, stem cells, cancer

## Abstract

Damage to normal human brain cells from exposure to ionizing radiation may occur during the course of radiotherapy or from accidental exposure. Delayed effects may complicate the immediate effects resulting in neurodegeneration and cognitive decline. We examined cellular and molecular changes associated with exposure of neural stem/progenitor cells (NSPs) to ^137^Cs γ-ray doses in the range of 0 to 8 Gy. Subventricular zone NSPs isolated from newborn mouse pups were analyzed for proliferation, self-renewal, and differentiation, shortly after irradiation. Strikingly, there was no apparent increase in the fraction of dying cells after irradiation, and the number of single cells that formed neurospheres showed no significant change from control. Upon differentiation, irradiated neural precursors did not differ in their ability to generate neurons, astrocytes, and oligodendrocytes. By contrast, progression of NSPs through the cell cycle decreased dramatically after exposure to 8 Gy (*p* < .001). Mice at postnatal day 10 were exposed to 8 Gy of γ rays delivered to the whole body and NSPs of the subventricular zone were analyzed using a four-color flow cytometry panel combined with ethynyl deoxyuridine incorporation. Similar flow cytometric analyses were performed on NSPs cultured as neurospheres. These studies revealed that neither the percentage of neural stem cells nor their proliferation was affected. By contrast, γ-irradiation decreased the proliferation of two classes of multipotent cells and increased the proliferation of a specific glial-restricted precursor. Altogether, these results support the conclusion that primitive neural precursors are radioresistant, but their proliferation is slowed down as a consequence of γ-ray exposure.

## Introduction

Head irradiation is an important adjuvant therapy for adult and pediatric patients with primary and metastatic brain tumors (Foote et al., 2010; Galloway et al., 2011; Jenkinson et al., 2011; MacDonald et al., 2011; Wiggenraad et al., 2011; Lyons et al., 2012). Although irradiation is effective in eradicating tumor cells and improving a patient’s prognosis when combined with other therapies, long-term brain cancer survivors who receive cerebral irradiation suffer neurobehavioral dysfunction, including cognitive and memory deficits (Lee et al., 1989; Abayomi et al., 1990; Crossen et al., 1994; Roman and Sperduto, 1995; Anderson et al., 2000; Surma-aho et al., 2001; Nagel et al., 2006; Saxe et al., 2006; Achanta et al., 2007; Aarsen et al., 2009; Calabrese and Schlegel, 2009). Delayed effects of head irradiation may arise from death of irradiated cells, persistent damage to cells that survive the radiation exposure, or depletion of progenitors thus impairing cell replacement. They may also arise from stressful effects that are propagated from irradiated to nonirradiated bystander cells, including neural stem cell/progenitors (NSPs) (Azzam et al., 2001; Ivanov and Hei, 2014). Hippocampal dysfunction is a prominent feature of postirradiation neuropsychological sequelae (Abayomi et al., 1990; Abayomi 2002; Nagel et al., 2006; Cortes-Wanstreet et al., 2009), and cognitive changes often appear as deficits in hippocampal functions of learning, memory, and spatial information processing (Abayomi et al., 1990; Abayomi 2002; Nagel et al., 2006; Saxe et al., 2006; Naylor et al., 2008). The hippocampus hosts neural precursors, and radiation damage to these cells is known to inhibit hippocampal neurogenesis. Among other factors, depletion of neural precursors was suggested to be a potential cause of radiation-induced cognitive impairment and other neurodegenerative outcomes (Roman and Sperduto, 1995; Siffert et al., 2000; Tada et al., 2000; Madsen et al., 2003; Mizumatsu et al., 2003; Saxe et al., 2006; Monje et al., 2007; Rola et al., 2007; Naylor et al., 2008; Fike et al., 2009; Acharya et al., 2010).

In the postnatal brain, neural precursors reside in the subgranular zone (SGZ) of the hippocampus and within the subventricular zones (SVZs) of the lateral ventricles (Gage, 2000). Although these neural precursors share the ability to proliferate, migrate, and differentiate into neurons and glia, they have different development potentials and sensitivities to ionizing radiation (Seaberg and van der Kooy, 2002; Romanko, Rola, et al., 2004; Romanko, Rothstein, et al., 2004; Hellstrom et al., 2009; Hellstrom et al., 2011). One day after exposure to a moderate dose (6 Gy) of high energy X rays, neural precursor proliferation was equally reduced in these two neural precursor niches (Hellstrom et al., 2009). Whereas hippocampal neurogenesis failed to recover, the SVZ NSPs recovered over a period of 2 months (Gage, 2000; Hellstrom et al., 2011). This was explained by the presence of a higher number of stem cells in the SVZ that are more resilient to irradiation injury and to a more favorable response of the SVZ microglia (Gage, 2000; Hellstrom et al., 2011). However, the molecular and intrinsic responses of the NSPs of the SVZ to radiation have not been clearly elucidated. Furthermore, several reports have shown that memory dysfunction occurs in the absence of any overt pathology (Dignam, 1990), suggesting a disruption of more subtle physiological processes (Lee et al., 2005).

Presently, there are limited data describing the behavior of neural stem cells (NSCs) and progenitors of the SVZ during recovery from radiotherapy. Much remains to be determined as to the direct effects of ionizing radiation on the survival, proliferation, self-renewal, and differentiation of NSPs. Understanding the factors involved in radiation sensitivity of neural precursors and the overall process of neurogenesis may provide key insights into strategies to prevent degenerative outcomes in the months and years after radiation exposure (Limoli et al., 2004; Acharya et al., 2009; Alwood et al., 2010). In this study, we investigated the cellular and molecular changes associated with exposure of NSPs from the SVZ to absorbed doses of ^137^Cs γ rays ranging from as low as 0.1 Gy to as high as 8 Gy.

## Materials and Methods

### Animals

C57BL/6 mice at postnatal day 7 (P7) and 10 (P10) were generated from breeders purchased from Jackson Laboratories (Bar Harbor, ME). All experiments were performed in accordance with research guidelines set forth by the Institutional Animal Care and Use Committee of the New Jersey Medical School.

### Animal Irradiation

Mice at postnatal day 10 were exposed to a mean absorbed dose of 8 Gy of ^137^Cs γ rays (3 Gy/min; linear energy transfer [LET] ∼ 0.9 keV/µm in liquid water) delivered to the whole body in a ventilated irradiator (J.L. Shepherd, Mark I, San Fernando, CA) located within the vivarium. The mice were placed in a multichamber device (a single mouse per chamber) on a rotating platform to ensure uniform exposure. Control mice were handled in parallel but were sham treated. The mice were equipped with individual dosimeters to verify the delivered dose (Mirion Technologies, Irvine, CA). The average absorbed dose was not statistically different among individual mice.

### Mouse Neurosphere Cultures

NSP cultures were prepared from newborn wild-type C57BL/6 mouse pups using previously described protocols (Brazel et al., 2005) that were optimized in our laboratories. Briefly, neonatal mice were euthanized by rapid decapitation and the dorsolateral aspect of the SVZ was microsurgically isolated, mechanically minced, and enzymatically dissociated to generate a single cell suspension. These cells were cultured in 35 mm diameter dishes in a defined growth medium supplemented with 20 ng/ml of epidermal growth factor (EGF) (PeproTech, Rocky Hill, NJ) and 10 ng/ml of fibroblast growth factor (FGF)-2 (PeproTech, Rocky Hill, NJ) with 1 ng/ml of heparin sulfate. The defined medium used contained 10 ng/ml d-biotin, 25 µg/ml insulin, 20 nM progesterone, 100 µM putrescine, 5 ng/ml selenium, 50 µg/ml apo-transferrin, 15 mM 4-(2-hydroxyethyl)-1-piperazineethanesulfonic acid (HEPES), and 50 µg/ml gentamycin sulfate in Dulbecco’s Modified Eagle’s medium:F12. The cells were fed every 2 to 4 days and maintained in a humidified incubator at 5% CO_2_ and 95% room air at 37℃. Cell passaging was accomplished by incubating primary spheres in Accutase (EMD Millipore, MA) for 5 min at 37℃ followed by mechanical dissociation of the spheres into a single cell suspension. These primary spheres were passaged to generate the cells to be used in experiments. As this population is comprised of 1% NSCs and a heterogeneous set of neural progenitors, we refer to these cells as neural/stem progenitors (Buono et al., 2012).

### Cells Destined for Irradiation

One day prior to irradiation, neurospheres were enzymatically and mechanically dissociated and seeded into 24-well plates at a density of 2.5 × 10^4^ cells/ml for neurosphere quantitation and differentiation studies. They were seeded at a density of 2.5 × 10^5^ cells/ml in 60-mm diameter dishes for multicolor flow cytometry and immunoblotting analyses. Exponentially growing cultures were either sham irradiated or exposed to γ rays (LET ∼ 0.9 keV/µm in liquid water) from a ^137^Cs source (J.L. Shepherd Mark I, San Fernando, CA). They were placed on a rotating platform and irradiated at dose rates that resulted in delivery of the specific desired dose in 2 to 3 min. The length of exposure ensured uniform irradiation of the whole cell population. Following irradiation, the cells were immediately reincubated at 37 ℃ until the time of assay.

### Neurosphere Survival

A single cell suspension of NSPs at second passage was seeded into 24-well tissue culture plates at a density of 2.5 × 10^4^ cells/ml. The cells were then exposed to γ-ray-absorbed doses ranging from 0 to 8 Gy. Neurospheres were quantified as described previously (Alagappan et al., 2009). Cells were propagated in medium supplemented with growth factors for 6 days at which point pictures were taken using an EVOS microscope (AMG, Bothell, WA) under ×10 or × 40 magnification. The number of spheres per well was determined and the radii of the spheres were measured with Image J (NIH, Bethesda, MD) and compared with nonirradiated controls. A minimum of 50 neurospheres per condition were randomly selected for analysis.

### Neurosphere Differentiation

Neurospheres arising from irradiated single cell suspensions were collected and resuspended at an approximate density of 100 to 200 spheres/ml. A total of 100 µl of the neurosphere suspension was then plated onto poly-d-lysine and laminin-coated chamber slides in growth medium containing 2% fetal bovine serum (FBS) without growth factors. After incubation for 16 hr, the medium was changed to a growth medium containing 0.5% FBS. The neurospheres were allowed to differentiate for 7 days, with media replenished every 2 days. Cells were stained using primary antibodies against glial fibrillary acidic protein (GFAP) (astroglia), TuJ1 (neurons), and O4 (oligodendrocytes), followed by fluorescence tagged secondary antibodies and counterstained with a 4′-6′ diamidino-2-phenylindole (DAPI; see below).

### Neurosphere Immunohistochemistry

After the differentiation period, the cells were washed twice with BCH solution consisting of 10% fetal calf serum in Minimal Essential Medium (MEM)/F12 medium with 4.8 mg/ml HEPES. They were reacted at room temperature for 45 min with O4 hybridoma culture supernatant diluted 1:5 in BCH supplemented with 10% lamb serum. After thorough rinsing with BCH, the cells were incubated for 45 min at room temperature in donkey anti-mouse IgM DyLight Red at 1:300 dilution (Jackson Immunoresearch, West Grove, PA). The cells were then fixed using 3% (vol/vol) paraformaldehyde (PFA) in phosphate-buffered saline (PBS) for 20 min at room temperature. The cells were rinsed with 100 mM glycine to quench PFA for 5 min and washed three times with PBS. Then, the cells were fixed with ice-cold methanol for 20 min at −20℃. After two rinses with tris-buffered saline (TBS), the cells were blocked in Tris-Goat serum-BSA (TGB) superblock (10% bovine serum albumin [BSA] in 0.05 M Tris buffer with 10% normal goat serum) overnight in a humidified environment at 4℃. The cells were then stained for 45 min at room temperature with anti-TuJ1 (1:500; Covance, Princeton, NJ) and anti-GFAP (1:500; Roche Products) in TGB diluent with 0.03% Triton-X-100. The cells were then incubated for 45 min at room temperature in goat anti-mouse DyLight488 IgG2a for TuJ1 (1:300; Jackson Immunoresearch) and goat anti-rabbit DyLight647 for GFAP (1:300; Jackson Immunoresearch). Finally, the cells were stained with DAPI (1:2500) for 5 min at room temperature followed by four rinses with TBS. The coverslips were mounted with Gel/Mount (Biomeda) and allowed to dry overnight in the dark. Immunoreactive cells were visualized using an Olympus Provis AX70 microscope and images of the cells were collected using a Q-imaging CCD camera interfaced with iVision scientific imaging software (Biovision, Exton, PA). Labeled cells in at least four random (nonadjacent) fields were counted per well under 20 ×or 40 ×magnification and a total of four wells per independent group were evaluated, with at least 100 cells counted based on DAPI staining. To evaluate the potentiality of a single spheroid, a minimum of 50 neurospheres per group were assessed for their potentiality. Spheres were categorized as either glial restricted (containing oligodendrocytes and astrocytes) or tripotential (containing neurons, astrocytes, and oligodendrocytes).

### Cell Cycle Analysis

One day prior to irradiation, neurospheres were enzymatically and mechanically dissociated and plated at a seeding density of 1 × 10^6^ cells per 60-mm diameter dish. Exponentially growing cultures of neural precursors were γ-irradiated and harvested at 4, 8, 24, and 48 hr postirradiation. The cultures were then dissociated into a single cell suspension and fixed with ice-cold 70% ethanol and stored at −20℃ until analyzed. On the day of assay, the cell samples were resuspended in PBS supplemented with 1% FBS. Following washing, the cells were stained in PBS supplemented with RNase (50 u/ml, Sigma-Aldrich, St. Louis, MO) and propidium iodide (PI, 10 µg/ml, Sigma-Aldrich) for 30 min at 37℃ and placed in the dark at room temperature. Subsequently, after passing through a cell strainer, the single cells were analyzed for DNA content by BD FACSCalibur flow cytometer (Franklin Lakes, NJ). A minimum of 10,000 cells were analyzed at each time point. Appropriate gating strategies were applied to exclude cell debris and aggregates. Flow cytometry data were analyzed using ModFit LT™ software (Verity Software House, Topsham, ME).

### Western Blotting

One day prior to irradiation, neurospheres were enzymatically and mechanically dissociated and plated at 1 × 10^6^ cells per 60-mm diameter dish. Exponentially growing cultures of neural precursors were γ-irradiated with various doses and harvested at indicated time points. The harvested cells were washed with ice-cold PBS and lysed in whole cell lysis buffer (50 mM Tris-HCl, pH 7.6, 50 mM NaCl, 0.1% sodium dodecyl sulfate [SDS], 1% Triton-X-100, and protease inhibitors; Lee et al., 2005). The lysates were further passed through a 26-gauge needle 10 times and clarified by centrifugation at 12,000 *g* at 4℃ for 10 min. Aliquots of 30 µg of protein of whole cell lysate were fractionated by 4 to 20% SDS-polyacrylamide gel electrophoresis and transferred to polyvinylidene fluoride membrane. The blot was reacted with anti-P-p53ser15 (Cell Signaling Technology, Danvers, MA), anti-p53 (Cell Signaling Technology), anti-proliferating cell nuclear antigen (PCNA; Santa Cruz Biotechnology, Santa Cruz, CA), and anti-Glyceraldehyde 3-phosphate dehydrogenase (GAPDH) (Sigma-Aldrich, St. Louis, MO) antibodies. Experiments were repeated three or four times.

### EdU Incorporation and Multicolor Flow Cytometry Analysis

Exponentially growing neurospheres were enzymatically and mechanically dissociated and plated at a seeding density of 1 × 10^6^ cells per 60-mm diameter dish 1 day prior to irradiation. They were γ-irradiated as described previously and then incubated in 10 µM ethynyl deoxyuridine (EdU; Life Technologies) overnight. To ensure a single cell suspension, the cells were dissociated with 0.2 Wünsch unit (WU)/ml of Liberase DH (Roche) and 250 µg of DNase1 (Sigma) in PGM solution (PBS with 1 mM MgCl_2_ and 0.6% dextrose) and then incubated in a 37℃ water bath for 5 min with gentle shaking. An equal volume of PGM was added and spheres were placed on a shaker (LabLine) at 220 rpm at 37℃ for 10 min. To analyze the *in vivo* responses of SVZ neural precursors to γ-irradiation, SVZs were isolated by microdissection and dissociated with 0.45 WU/ml of Liberase DH and 250 µg of DNase1 in PGM with shaking at 220 rpm at 37℃ for 30 min. After enzymatic digestion, Liberase DH was quenched with 10 ml of PGB (PBS without Mg^2+^ and Ca^2+^ with 0.6% dextrose and 2 mg/ml fraction V of BSA) and cells were centrifuged for 5 min at 200 ×*g*. Cells were dissociated by repeated trituration, collected by centrifugation, counted using ViCell (Beckman Coulter, Miami, FL) and diluted to at least 10^6^ cells per 50 µl of PGB. Staining was performed in 96 V-bottom plates using 150 µl of volume. For surface marker analysis, cells were incubated in PGB for 25 min with antibodies against Lewis-X-v450 (1:20, LeX/CD15, MMA; BD Bioscience), CD133-APC (1:50,13A4; eBioscience), CD140a (1:400, APA5; BioLegend), and NG2 chondroitin sulfate proteoglycan (1:50, AB5320; Millipore). Cells were washed with PGB by centrifugation at 300 × *g*. Goat anti-rabbit IgG Alexa Fluor 700 (1:100; Invitrogen) was used for NG2. Secondary antibodies and DAPI (Sigma) were incubated with cells in PGB for 20 min. DAPI was used to exclude dead cells. Cells were washed with PGB by centrifugation at 300 ×*g*. Cells from the SVZ were fixed with 1% ultrapure formaldehyde (50,000; Polysciences, Inc) for 20 min, collected by centrifugation for 9 min at 609 ×*g*, resuspended in PBS w/o Mg^2+^ and Ca^2+^. They were subsequently permeabilized and incorporated EdU was stained with Click-iT reagent (Life Technologies) following manufacturer’s instruction. All sample data were collected on the BD LSR II (BD Biosciences Immunocytometry Systems). Appropriate gating strategies based on isotype controls were applied to exclude cell debris, aggregates, and nonspecific staining. Data were analyzed with FlowJo software (Tree Star, Inc).

### Flow Cytometry Analysis of SVZ Cells Irradiated In Vivo

At 20 hr and 22 hr after irradiation, 100 mg/kg EdU dissolved in PBS was injected intraperitoneally. Two hours later (i.e., 24 hr after irradiation), the mice were euthanized by carbon dioxide inhalation. The SVZs were isolated by microdissection and dissociated with 0.5 WU/ml of Liberase DH and 250 µg/ml of DNaseI in PGM with agitation at 220 rpm at 37℃ for 1 hr. Liberase DH was quenched with 10 ml of PGB and cells were centrifuged for 5 min at 300 × *g*. Cells were dissociated by repeated trituration, then filtered through a 100 µm cell strainer, washed in 25 ml PGB, and collected by centrifugation. Cells were resuspended in PGM and processed for flow cytometry as described previously.

### Statistical Analysis

Results of cell survival, as evaluated by neurosphere formation, cell differentiation, and the redistribution of cells at the different stages of the cell cycle were analyzed by two-way analysis of variance (ANOVA) or Kruskal–Wallis test followed by appropriate post hoc tests. Statistical tests were conducted using GraphPad InStat (GraphPad Software, La Jolla, CA).

## Results

### Ionizing Radiation Inhibits Proliferation of Neural Precursor Cells Without Affecting Their Survival or Long-Term Self-Renewal

**Figure 1.**
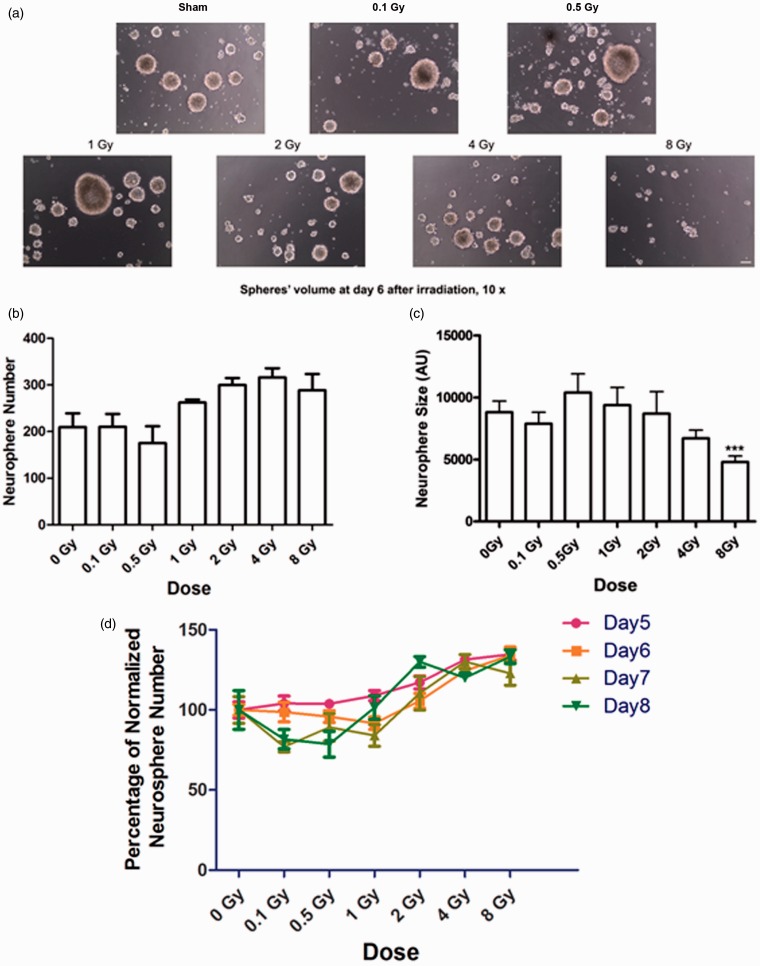
**Exposure to high and low doses of ^137^Cs γ rays does not affect survival or self-renewal of neural stem/progenitors from the SVZ but reduces their proliferation.** A single cell suspension of NSPs at second passage was exposed to γ rays in a dose range from 0 to 8 Gy and then seeded into 24-well tissue culture plate wells at 2.5 × 10^4^ cells/ml. The cells were then propagated in a biochemically defined growth medium supplemented with 20 ng/ml EGF and 10 ng/ml FGF-2 for 6 days in a humidified incubator maintained at 5% CO_2_ and 95% room air at 37℃. (a) Images of representative neurospheres after sham treatment or exposure to γ rays. Photographs were taken at 10 ×. Scale bar is 10 µm. (b) Average number of spheres formed per well after irradiation (*n* = 8) at indicated doses. There was no significant change in the number of neurospheres after irradiation compared with control (0 Gy; *p* > .05 by ANOVA). (c) Average size of neurospheres after irradiation at indicated doses (*n* > 50/group). After exposure to 8 Gy, the size of neurospheres decreased significantly (*p* < .001 by ANOVA). *p* values were determined by Kruskal–Wallis test followed by Dunn’s multiple comparisons test compared with 0 Gy. (d) Plot of neurosphere abundance normalized to control over radiation doses. There was no significant change in the number of neurospheres from 5 to 8 days after irradiation (*p* > .05 by repeat ANOVA). Bars represent averages ± SEM. Data represent averages of the three independent experiments. SVZ = subventricular zone; NSPs = neural stem/progenitor cells; ANOVA = analysis of variance; EGF = epidermal growth factor; FGF = fibroblast growth factor.

To investigate the effects of ionizing radiation on NSP survival, self-renewal, and proliferation, we evaluated the number and volume of neurospheres following exposure to acute ^137^Cs γ rays. Neurosphere number reflects the self-renewal of NSCs, a cardinal feature of these cells. On the other hand, sphere volume is an index of proliferation (i.e., the size of the sphere reflects the proliferation of the initial sphere-forming cells and their descendants). On day 6 after irradiation, quantification of the number of spheres that formed revealed no significant change in the number of spheres formed by irradiated NSPs compared with sham-irradiated cells (*p* > .05; Figure 1(a) to (c)). Similar results were observed when spheres were evaluated on days 5, 7, and 8 after irradiation (Figure 1(d)). These results indicate that exposure to ionizing radiation did not adversely affect the ability of a NSP to produce a subsequent neurosphere, meaning that its self-renewal was not impaired. However, at radiation doses larger than 0.5 Gy, there was a dose-dependent reduction of average neurosphere volume. Following exposure to 8 Gy, this reduction in average neurosphere volume reached statistical significance compared with sham-irradiated neurospheres (4795 ± 483.4 AU vs. 8802 ± 908.3 AU, *p* < .0001; Figure1(c)).

### Ionizing Radiation Does Not Change the Differentiation Pattern of Neural Precursor Cells

**Figure 2.**
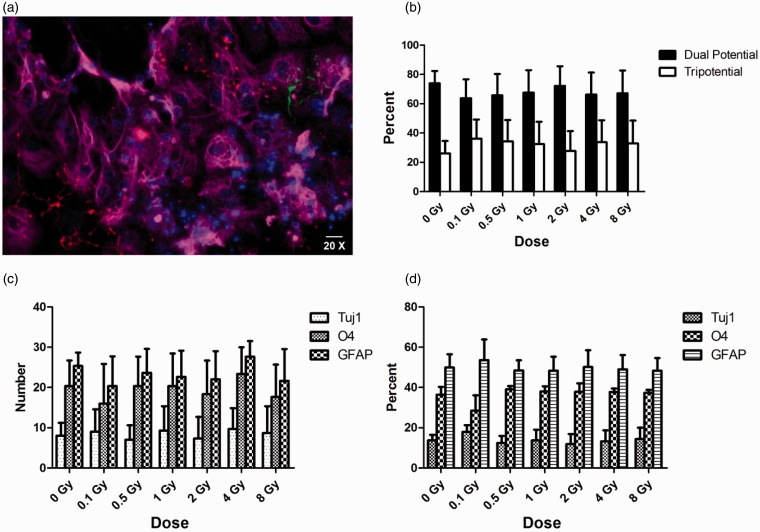
**Exposure to ^137^Cs γ rays does not change potency of NSPs derived from the SVZ.** Neurospheres were differentiated for 7 days after exposure to γ rays in a dose range from 0 to 8 Gy. Spheres formed from irradiated cells were seeded onto poly-l-lysine and laminin-coated chamber slides in growth medium containing 2% FBS. After 16 hr, the medium was changed to a growth medium containing 0.5% FBS. Cells were stained using antibodies against GFAP (astroglia), TuJ1 (neurons), and O4 (oligodendrocytes). Cells were evaluated by fluorescence microscopy (Olympus Optical, Tokyo, Japan). (a) Representative image of neurosphere stained for GFAP (Magenta), TuJ1 (Green), O4 (Red), and DAPI-positive nuclei (Blue). Scale bar is 10 µm. (b) Average percentage of neurospheres that were classified as glial restricted and tripotential. (c) Average number of neurospheres expressing different makers after irradiation at indicated doses. (d) Average percentages of cells expressing different makers after irradiation at indicated doses. There was no significant change in the potency of neurospheres after irradiation compared with sham-irradiated cells (*p* > .05 by ANOVA; (*n* = 3). *p*-values were determined by ANOVA followed by Dunn’s multiple comparisons test compared with 0 Gy. Error bars indicate SEM. Data represent averages of the three independent experiments. SVZ = subventricular zone; NSPs = neural stem/progenitor cells; DAPI = 4′-6′ diamidino-2-phenylindole; FBS = fetal bovine serum; ANOVA = analysis of variance.

Six days after irradiation, primary cultures were differentiated for 7 days to evaluate the effect of γ rays on the multipotentiality of the neural precursors. Cells were reacted with markers for astrocytes (GFAP), neurons (TuJ1), and oligodendrocytes (O4). Nuclear counterstaining was performed with DAPI (Figure 2(a)). The colonies of cells produced by the neurospheres were categorized into two groups: glial restricted or tripotential. Even though the spheres were smaller, reflecting reduced number of progenitors, there was no significant difference in the fraction of tripotential- or glial-restricted colonies in the irradiated groups compared with sham-irradiated cells (Figure 2(a) to (c)). Furthermore, when the percentages of neurons, astrocytes, and oligodendrocytes were quantified, there were no shifts in the relative proportions of each cell type (Figure 2(d)).

### Inhibition of DNA Synthesis and Cell Cycle Arrest of High-Dose γ-Irradiated Neural Precursor Cells

**Figure 3.**
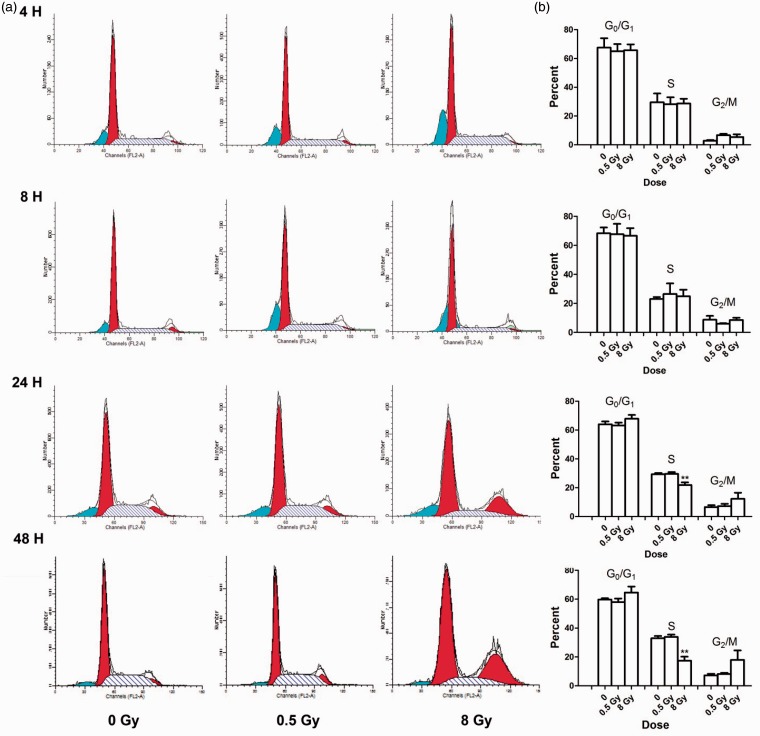
**Exposure to high doses of ^137^Cs γ rays perturbs progression through the cell cycle of NSPs derived from the SVZ**. A single cell suspension of neural stem cell and progenitors was exposed to γ rays at 0, 0.5, and 8 Gy. Cells were fixed, stained with PI, and cell cycle analysis was performed at various times after irradiation. (a) Representative histograms from four individual experiments illustrate the distribution of cells in the phases of the cell cycle based on DNA content. (b) Quantitation of cell cycle analyses. After 24 and 48 hr of high-dose irradiation, a significant proportion of the cells were arrested in G2/M. Statistical analysis showed that 8 Gy of gamma rays also inhibited cell cycle progression at S phase after 24 hr (*n* = 4) and 48 hr (*n* = 3). **p < .01 by ANOVA followed by Dunnett’s multiple comparisons test compared with 0 Gy. Error bars indicate SEM. Data represent averages of the four independent experiments. SVZ = subventricular zone; NSPs = neural stem/progenitor cells; PI = propidium iodide; ANOVA = analysis of variance.

We examined the effects of ionizing radiation on the progression through the cell cycle of NSPs derived from neurospheres at 4, 8, 24, and 48 hr after exposure to γ rays (Figure 3). Cell cycle analyses revealed significant inhibition of DNA synthesis 24 hr after exposure to 8 Gy. At 4 hr after exposure to 0.5 or 8 Gy, there was an increase in the number of irradiated NSPs in G2/M phase compared with sham-irradiated control cells. By 24 hr after irradiation with 8 Gy, there was a 26% reduction in the number of irradiated cells compared with sham-irradiated cells (*p* < .01). In parallel, there was an 86% increase in the number of irradiated cells in G2/M phase compared to sham-irradiated cells. Similar results were observed 48 hr after irradiation; the number of irradiated NSPs was reduced by 54% in S phase compared with sham-irradiated cells (*p* < .01). Moreover, there was a greater than twofold decrease in the number of irradiated NSPs in S phase, which was paralleled by a greater than twofold increase in the number of NSPs in G2/M phase. This suggested that exposure of NSPs in the SVZ to an absorbed dose of 8 Gy caused DNA synthesis inhibition in S phase and cell cycle arrest in G2/M phase (Figure 3). We also evaluated increases in apoptosis by measuring the sub-G1 peak; however, no significant change in sub-G1 peak was observed in irradiated cells compared with controls (Figure 3).

**Figure 4.**
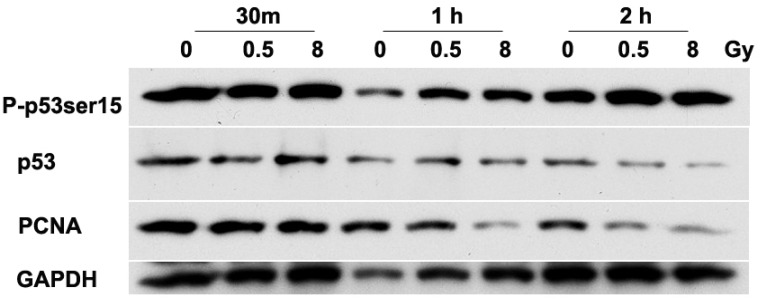
**Exposure of NSPs to ^137^Cs γ rays upregulates p53 and decreases total levels of PCNA**. Cell lysates were prepared from neural precursors after 0, 0.5, and 8 Gy of irradiation at 3 time points and analyzed by Western Blot; 30 ng of protein was loaded per lane. Compared with sham-irradiated NSPs, p53 induction was detectable by 30 min after irradiation and returned to baseline by 2 hr. The increase in level of P-p53ser15 (a marker of DNA damage) persisted for at least 2 hr after irradiation. Reductions in PCNA levels were detected within 1 hr after exposure to 8 Gy and within 2 hr after exposure to 0.5 Gy. Representative blots of the three individual experiments are shown. NSPs = neural stem/progenitor cells; PCNA = proliferating cell nuclear antigen.

We analyzed the expression of p53 and PCNA in irradiated NSPs, as these proteins are implicated in control of cell cycle progression (Figure 4). The phosphorylation at ser15 of p53, a DNA damage marker, was clearly increased by 30 min to 1 hr after irradiation with 0.5 and 8 Gy. By 2 hr, P-p53ser15 returned to baseline level (Figure 4). The increases in P-p53ser15 were associated with increases in p53 native level that were equally detectable by 30 min after exposure to either 0.5 or 8 Gy. The latter data are consistent with stabilization of p53 and activation of signaling pathways that regulate cell cycle checkpoints as was described in several mammalian cell types exposed to DNA damaging agents (Azzam et al., 2000; Zhou and Elledge, 2000). The radiation dose-dependent decreases in the levels of PCNA observed at 1 hr after exposure to 8 Gy further support the cell cycle delays described in Figure 4. The absence of significant effect on cell cycle progression in cells exposed to 0.5 Gy was also reflected in attenuated decreases in the level of PCNA. The levels of PCNA were unchanged in cells harvested for analysis at 1 hr after exposure to 0.5 Gy but were reduced by 2 hr.

### Ionizing Radiation Affects the Proliferation of Neural Precursors, Without an Apparent Effect on the NSCs

To more accurately assess the effects of ionizing radiation on NSCs and progenitors of the SVZ, we further phenotyped the subsets of SVZ cells comprising *in vitro* neurosphere cultures and cells of the SVZ of irradiated mice using multicolor flow cytometry (Tables 1–6). EdU incorporation was evaluated to assess the effects of irradiation on inhibition of proliferation. EdU is a nucleoside analog of thymidine and is incorporated into DNA during active DNA synthesis as a newer alternative to 5-bromo-2′-deoxyuridine to evaluate the S-phase checkpoint of the cell cycle (Buck et al., 2008; Salic and Mitchison, 2008). After irradiation, there was no significant change in total percentage of CD133+/LeX+/NG2-/CD140a- NSCs in both *in vitro* and *in vivo* studies compared with nonirradiated control. Interestingly, the *in vitro* and *in vivo* studies showed different abundance patterns of other progenitor cells. *In vitro*, irradiation decreased the total percentage of CD133-/LeX+/NG2+/CD140a+, Platelet-derived growth factor (PDGF)/FGF responsive multipotential progenitors (PFMPs; Table 1). Whereas, *in vivo* irradiation decreased total CD133-/LeX+/NG2-/CD140a-multipotential progenitors (MP1), it increased total CD133-/LeX+/NG2+/CD140a-bipotential neuronal and astrocytic associated progenitors-/glial-restricted progenitors (BNAPs/GRP1s; Table 4). After EdU gating was applied to cells cultured *in vitro*, most proliferating progenitors that incorporated EdU were decreased except a subset of CD133-/LeX-/NG2+/CD140a+ glial-restricted progenitors (GRP3s), which was increased (Table 3). EdU positive BNAP/GRP1s showed no change. Meanwhile, after *in vivo* administration of EdU, the fractions of EdU positive MP1s and CD133+/LeX+/NG2+/CD140a-MP2s were decreased by irradiation, but BNAP/GRP1 and GRP3 EdU incorporation was increased by irradiation (Table 6). *In vitro*, high-dose irradiation reduced overall EdU incorporation (56.65 ± 2.03% vs. 32.84 ± 3.39%, *n  *= 3), which may reflect slowing down of proliferation (Table 2). Low-dose irradiation also reduced EdU incorporation but resulted in no significant change (56.65 ± 2.03% vs. 43.35 ± 3.71%, *n* = 3). *In vivo*, irradiation also decreased total EdU incorporation, but this trend did not reach statistical significance (10.33 ± 1.13% vs. 7.9 ± 1.02%, *n* = 4; Table 5).

**Table 1.** Frequency of Neural Progenitors From the SVZ After *In Vitro* Exposure to ^137^Cs γ Rays.

*In vitro*: Total cells (normal panel without EdU gating)								
Treatment group	NSC	MP1	MP2	MP3/GRP2	PFMP	BNAP/GRP1	GRP3	Other
	CD133+	CD133−	CD133+	CD133−	CD133−	CD133−	CD133−	
	LeX+	LeX+	LeX+	LeX−	LeX+	LeX+	LeX−	
	NG2−	NG2−	NG2+	NG2+	NG2+	NG2+	NG2+	
	CD140a−	CD140a−	CD140a−	CD140a−	CD140a+	CD140a−	CD140a+	
0 Gy	0.34%	11.24%	5.95%	42.31%	5.65%	1.91%	27.14%	5.48%
	±0.12	±1.47	±0.59	±0.89	±0.50	±0.08	±1.78	±1.77
0.5 Gy	0.32%	7.29%	6.36%	48.57%	3.03%*	1.95%	29.41%	3.08%
	±0.05	±1.25	±0.81	±0.90	±0.09	±0.20	±1.91	±1.08
8 Gy	0.26%	6.96%	4.56%	46.33%	2.47%*	2.28%	33.68%	3.46%
	±0.07	±1.1	±0.32	±2.85	±0.26	±0.25	±1.64	±1.48

*Note*. MP = multipotential progenitor; PFMP = PDGF/FGF responsive multipotential progenitors; GRP = glial-restricted progenitor; BNAP = bipotential neuronal and astrocytic associated progenitor; ANOVA = analysis of variance; EdU = ethynyl deoxyuridine; NSPs = neural stem/progenitor cells; SVZ = subventricular zone. A single cell suspension of NSPs was exposed to γ rays at 0, 0.5, and 8 Gy. Cells were analyzed by flow cytometry for CD133, LeX, CD140a, and NG2. *p < .05 by ANOVA followed by Dunnett’s multiple comparisons test compared with 0 Gy.

* *p* < .05 vs. 0 Gy, *n* = 3.

**Table 2.** Frequency of Proliferating Cells After Exposing *In Vitro* Neural Progenitors From the SVZ to ^137^Cs γ Rays.

*In vitro*: Total EdU+ cells	
Treatment group	Total EdU+ cells
0 Gy	56.65 ± 2.03%
0.5 Gy	43.35 ± 3.71%
8 Gy	32.84 ± 3.39%*

*Note*. SVZ = subventricular zone; NSPs = neural stem/progenitor cells; EdU = ethynyl deoxyuridine; ANOVA = analysis of variance. A single cell suspension of NSPs was exposed to γ rays at 0, 0.5, and 8 Gy. Prior to harvest, cells were incubated with 10 µM EdU overnight and then the cells were analyzed by flow cytometry and processed using Click-It chemistry for EdU. Data are averaged from three separate experiments. A *p < .05 by ANOVA followed by Dunnett’s multiple comparisons test compared with 0 Gy.

**Table 3.** Proliferation and Frequency of NSPs Derived From the SVZ After *In Vitro* Exposure to ^137^Cs γ Rays.

*In vitro*: Percentage of EdU+ cells								
Treatment group	NSC	MP1	MP2	MP3/GRP2	PFMP	BNAP/GRP1	GRP3	Other
	CD133+	CD133−	CD133+	CD133−	CD133−	CD133−	CD133−	
	LeX+	LeX+	LeX+	LeX−	LeX+	LeX+	LeX−	
	NG2−	NG2−	NG2+	NG2+	NG2+	NG2+	NG2+	
	CD140a−	CD140a−	CD140a−	CD140a−	CD140a+	CD140a−	CD140a+	
	EdU+	EdU+	EdU+	EdU+	EdU+	EdU+	EdU+	
0 Gy	0.01%	2.54%	5.24%	42.01%	2.62%	0.60%	12.40%	34.58%
	±0.01	±0.72	±0.81	±2.23	±0.54	±0.31	±1.27	±3.39
0.5 Gy	0.01%	0.85%*	4.93%	37.42%	1.07%	0.39%	12.77%	42.40%
	±0.01	±0.14	±1.00	±2.22	±0.24	±0.25	±1.20	±4.42
8 Gy	0.02%	0.41%*	1.00%*^	31.71%*	0.53%*	0.41%	28.1%*^	49.54%
	±0.02	±0.21	±0.29	±1.51	±0.29	±0.18	±4.51	±7.43

*Note*. MP = multipotential progenitor; PFMP = PDGF/FGF responsive multipotential progenitors; GRP = glial-restricted progenitor; BNAP = bipotential neuronal and astrocytic associated progenitor; NSPs = neural stem/progenitor cells; SVZ = subventricular zone; EdU = ethynyl deoxyuridine; ANOVA = analysis of variance. A single cell suspension of NSPs was exposed to γ rays at 0, 0.5, and 8 Gy. Prior to harvest, cells were incubated with 10 µM EdU overnight and then the cells were analyzed by flow cytometry. Data are averaged from three separate experiments. A *p < .05 or ^*p *< .05 by ANOVA followed by Dunnett’s multiple comparisons test compared with 0 Gy or 0.5 Gy**.**

* *p* < .05 vs. 0 Gy; ^*p* < .05 vs. 0.5 Gy, *n* = 3.

**Table 4.** Frequency of Neural Progenitors After *In Vivo* Exposure to ^137^Cs γ Rays.

*In vitro*: Percentage of EdU+ cells								
Treatment group	NSC	MP1	MP2	MP3/GRP2	PFMP	BNAP/GRP1	GRP3	Other
	CD133+	CD133−	CD133+	CD133−	CD133−	CD133−	CD133−	
	LeX+	LeX+	LeX+	LeX−	LeX+	LeX+	LeX−	
	NG2−	NG2−	NG2+	NG2+	NG2+	NG2+	NG2+	
	CD140a−	CD140a−	CD140a−	CD140a−	CD140a+	CD140a−	CD140a+	
Nonirradiated control	2.27%	5.60%	3.66%	47.30%	3.26%	3.79%	2.33%	31.79%
	±0.67	±0.42	±0.65	±8.75	±1.01	±2.71	±0.35	±9.89
Irradiated	1.76%	1.79%*	3.73%	44.63%	3.57%	8.44%*	4.07%	32.01%
	±0.99	±0.23	±0.72	±4.09	±1.41	±2.15	±1.41	±7.01

*Note*. MP = multipotential progenitor; PFMP = PDGF/FGF responsive multipotential progenitors; GRP = glial-restricted progenitor; BNAP = bipotential neuronal and astrocytic associated progenitor; SVZ = subventricular zone; EdU = ethynyl deoxyuridine. Mice were exposed to 0 or 8 Gy of γ rays. SVZs from irradiated mice were dissected, prepared, and analyzed with multicolor flow cytometry using CD133, LeX, CD140a, and NG2 and processed using Click-It chemistry for EdU. Data are averaged from four animals per group. *p < .05 by unpaired *t* test. Error bars indicate SEM.

* *p* < .05 vs. nonirradiated control, *n* = 4.

**Table 5.** Frequency of Proliferating Cells After Exposing Neural Progenitors *In Vivo* to ^137^Cs γ Rays.

*In vivo*: Total EdU+ cells	
Treatment group	Total EdU+ cells
Nonirradiated control	10.33 ± 1.13%
Irradiated	7.90 % ± 1.02%

EdU = ethynyl deoxyuridine; SVZs = subventricular zones.

Mice were exposed to 0 or 8 Gy of γ rays. At 20 hr and 22 hr after irradiation, 100 mg/kg EdU was injected intraperitoneally. Two hours later, the animals were euthanized and the SVZs were dissected, prepared, and analyzed with multicolor flow cytometry for EdU. Data are averaged from four animals per group. Error bars indicate SEM.

**Table 6.** Proliferation and Frequency of NSPs From the SVZ After *In Vivo* Exposure to ^137^Cs γ Rays.

*In vivo*: Total EdU+ cells								
Treatment group	NSC	MP1	MP2	MP3/GRP2	PFMP	BNAP/GRP1	GRP3	Other
	CD133+	CD133−	CD133+	CD133−	CD133−	CD133−	CD133−	
	LeX+	LeX+	LeX+	LeX−	LeX+	LeX+	LeX−	
	NG2−	NG2−	NG2+	NG2+	NG2+	NG2+	NG2+	
	CD140a−	CD140a−	CD140a−	CD140a−	CD140a+	CD140a−	CD140a+	
	EdU+	EdU+	EdU+	EdU+	EdU+	EdU+	EdU+	
Nonirradiated control	0.49%	4.34%	7.58%	67.75%	0.56%	3.20%	1.88%	14.20%
	±0.17	±0.99	±1.69	±4.02	±0.01	±0.79	±0.20	±2.79
Irradiated	0.64%	0.34%*	3.65%*	65.53%	0.34%	10.74%*	6.14%*	12.63%
	±0.10	±0.11	±0.82	±4.66	±0.06	±2.74	±1.17	±3.47

*Note*. MP = multipotential progenitor; PFMP = PDGF/FGF responsive multipotential progenitors; GRP = glial-restricted progenitor; BNAP = bipotential neuronal and astrocytic associated progenitor; NSPs = neural stem/progenitor cells; SVZs = subventricular zones; EdU = ethynyl deoxyuridine.

Mice were exposed to 0 or 8 Gy of γ rays. At 20 hr and 22 hr after irradiation, 100 mg/kg EdU was injected intraperitoneally. Two hours later the animals were euthanized and the SVZs were isolated, dissected, prepared, and analyzed with multicolor flow cytometry using CD133, LeX, CD140a, and NG2. They were processed using Click-It chemistry for EdU. Data are averaged from four animals per group. *p < .05 by unpaired *t*-test. Error bars indicate SEM.

* *p *< .05 vs. nonirradiated control, *n* = 4.

## Discussion

The salient findings in our study are threefold. First, NSCs derived from the SVZ appear inherently radioresistant, whereas neural progenitors are more radiosensitive. There was no significant difference in NSCs derived from the SVZ for the end points of abundance, immediate self-renewal, or differentiation potential when exposed to either low (0.5 Gy) or relatively high (8 Gy) doses of ^137^Cs γ rays (Figures 1 and 2 and Tables 1 and 3). Second, exposure to an absorbed dose of 8 Gy of γ rays impaired their ability to progress through the cell cycle (Figure 3; Tables 2 and 4). Specifically, the radiation inhibited DNA synthesis and arrested the cells in G2/M phase. Third, altered progression in the cell cycle was associated with regulation of p53 and PCNA (Figure 4, Tables 2–5). The observed decrease in PCNA level and increase in p53 level coupled with its phosphorylation in serine 15 are indicative of DNA damage that triggers cell cycle checkpoints, which ensure that the cell cycle progresses without any DNA damage, hence maintaining genomic integrity of surviving irradiated NSPs (Little, 1994).

Although the effects of ionizing radiation on neurogenesis in the adult hippocampus have been widely investigated (Peissner et al., 1999; Monje et al., 2002; Mizumatsu et al., 2003; Monje and Palmer, 2003; Monje et al., 2003; Limoli et al., 2004; Monje et al., 2007), few studies have analyzed the radiation sensitivity of SVZ neural precursors. Hellstrom et al. (2011) compared the differential recovery of NSCs in the SVZ and dentate gyrus after exposure to ionizing radiation (Hellstrom et al., 2009; Hellstrom et al., 2011). They demonstrated superior recovery of NSPs from the SVZ compared with hippocampal NSPs. This was attributed to a more supportive response of irradiated SVZ microglia to neurogenesis (Hellstrom et al., 2011). By contrast, it has been argued that the hippocampus does not contain stem cells but harbors more restricted progenitor cells than the SVZ. Seaberg and van der Kooy (2002) and Bull and Bartlett (2005) reported that the precursors in the hippocampus are lineage-restricted progenitors and not stem cells (Seaberg and van der Kooy 2002; Bull and Bartlett 2005; Seaberg et al., 2005). Furthermore, their data indicated that there are two discrete populations of progenitors in the SGZ, one giving rise to neurons, while the other giving rise to glial cells. This concept has been advanced to explain the higher sensitivity to ionizing radiation of hippocampus neural precursors compared with SVZ cells (Hellstrom et al., 2011; Seaberg et al., 2011).

Several studies have shown that NSCs are resistant to death stimuli. They are relatively resistant to death following neonatal hypoxia–ischemia (Levison et al., 2002; Romanko et al., 2007) and they are also resilient to glutamate, glycine, and muscimol toxicity *in vitro* (Brazel et al., 2005). Indeed, we recently demonstrated that postnatal rat NSPs express low levels of proapoptotic molecules and resist PI3K and ERK1/2 inhibition when compared with late oligodendrocyte progenitors. Furthermore, we found that a 72-hr treatment with inhibitors of PI3K and ERK1/2 signaling, eliminated lineage-restricted precursors but enriched for multipotential, self-renewing Nestin^+^/SOX-2^+^ precursors (Brazel et al., 2014). Interestingly, NSCs require high endogenous levels of reactive oxygen species (ROS) to self-renew (Le Belle et al., 2011). Together, these studies support the concept that NSCs possess inherent adaptation mechanisms that provide them with a survival advantage over other cells types when challenged by endogenous or exogenous oxidative stresses. This appears consistent with their critical role towards long-term healthy survival, which requires constant replacement and regeneration of progenitors subjected to oxidizing chemical species generated by oxidative metabolism or exposure to environmental oxidizing or other harmful factors. Extending the evidence that primitive neural precursors are resistant to death stimuli, here we show that exposure to absorbed doses, as high as 8 Gy of ^137^Cs γ rays, did not affect the immediate self-renewal properties of these cells (however, we cannot exclude the possibility that their long-term self-renewal might have been diminished). Our flow cytometry data support the conclusion that the NSCs are more resilient than progenitors, as there was no change in the percentage of CD133+/LeX+/CD140a-/NG2-cells, although there were decreases in the percentages of several multipotential progenitors. Clearly, additional experiments investigating cell death by annexin V staining, caspase activation, and poly(ADP ribose) polymerase cleavage will further shed light on the radiation sensitivity of NSPs in our studies.

Whereas there was no apparent effect of 0.5 or 8 Gy on cell death (as illustrated by the absence of a significant change in the number of cells derived from neurospheres in the sub-G_1_ phase after exposure to a low or high doses of γ rays), the proliferation of the precursors was affected. One index of proliferation is the average neurosphere size. When SVZ-derived NSPs were irradiated with a dose greater than 0.5 Gy, there was a dose-dependent decrease in the average size of spheres; although, the decrease was significant only at the highest dose of 8 Gy. Strikingly, after exposure to 0.5 Gy, the average neurosphere size was larger than control, albeit their numbers were the lowest among the other irradiated groups and the control neurospheres, although the changes in numbers did not reach statistical significance (Figure 1). These findings suggest a differential effect of low and high doses of γ rays on SVZ NSPs.

Similar results on the differential effects of low- and high-dose ionizing radiation on NSP proliferation were reported by Isono et al. (Isono et al., 2012). They showed that low doses of ionizing radiation temporarily arrested the proliferation of mouse neural precursors derived from embryonic stem cells, while cells irradiated at high doses permanently lost their proliferation capability (Isono et al., 2012). These differential effects at low- and high-dose radiation exposures are relevant to radiation protection issues as a greater number of individuals are being exposed to low-dose ionizing radiation during diagnostic radiography or occupational activities (de Toledo et al., 2006; Zhang et al., 2012).

Irradiation exerts direct action on all macromolecular targets (Little, 1994; Agarwal et al., 1995; Achanta et al., 2007; Tsuboi et al., 2007; Acharya et al., 2010; Azzam et al., 2012). Among the targets, damage to DNA integrity is critical and elicits a global stress response that is characterized by activation of an array of signaling pathways. Genes/proteins implicated in DNA repair, cell cycle checkpoints, apoptosis, and changes in gene expression are modulated in a dose-dependent manner by ionizing radiation (de Toledo et al., 1998; Amundson and Fornace, 2001; Budworth et al., 2012; Chaudhry et al., 2012). One of the most important regulatory events that is activated by ionizing radiation is the activation of the tumor suppressor protein p53, a critical transcriptional regulator of cell cycle progression (Agarwal et al., 1995; de Toledo et al., 1998; Ljungman, 2000). In our study, we observed increased level of p53 that was associated with enhanced phosphorylation of the protein on serine 15 in cells exposed to low- or high-dose γ rays. These results indicate that NSPs have sustained DNA damage as the specific phosphorylation on ser15 in p53 occurs in cells that suffer DNA double strand breaks and other DNA oxidative lesions (Canman et al., 1998). The increase in the native form of p53 reflects activation of signal transduction leading to stabilization of p53, which could be detected in our study by 30 min after irradiation. However, cell viability and DNA content assay showed that there was no significant increase in cell death after exposure to high dose of γ ray.

Stem cell populations possess an enhanced resistance to oxidative stress-mediated cell death (Romanko, Rothstein, et al., 2004; Madhavan et al., 2006, 2008). Proliferative NSCs with high endogenous levels of ROS were highly responsive to ROS-stimulated self-renewal and neurogenesis in a PI3K/Akt-dependant manner, which was identified as a protective mechanism to prevent excessive ROS from being generated (Le Belle et al., 2011). A recent study suggested that stem cells possess unique metabolic and proliferative properties that minimize insults to genomic integrity, which may be compromised by robust activation of DNA damage response in the long run (Mandal et al., 2011). The reduction in PCNA expression is consistent with induction of cell cycle checkpoints in irradiated NSPs. This was confirmed by the observed inhibition of DNA synthesis and G_2_/M arrest at 24 hr after exposure to 8 Gy (Figure 3). Further analyses of p53 downstream effectors (e.g., the cyclin/cyclin-dependent kinase inhibitor p21^Waf1^) may provide a basis for the unexpected absence of a G_1_ checkpoint in irradiated SVZ NSPs (Figure 2). The arrest in G_2_ phase of the cell cycle is consistent with the reduced proliferation of NSPs after exposure to 8 Gy. Similar results were obtained by Kato et al. (2007) in studies of early radiation responses of neural precursors derived from mice at embryonic day 14.5. They observed a G_2_ arrest by 12 hr after exposure to 2 Gy, which correlated with cdc2 phosphorylation pattern that is consistent with G_2_ arrest. Similar to our results, they also observed a dose-dependent suppression of proliferation of irradiated cells (Kato et al., 2007).

Prolonged inhibition of cell proliferation after radiation exposure has also been reported in the SVZ and dentate gyrus (Tada et al., 1999; Tada et al., 2000; Monje et al., 2002). A rapid activation of ataxia telangiectasia mutated (ATM) pathway leading to p53 phosphorylation and transcriptional activation of *WAF1* together with concomitant G_2_ cell cycle arrest was reported by Filion et al. (2009), when they irradiated pluripotent human embryonic stem cells with 5 Gy (Filion et al., 2009). Furthermore, Muthna et al. (2010) studied the effect of radiation doses ranging from 2 to 20 Gy on adult human dental pulp stem cells proliferative potential and the associated molecular pathways. Similar to our observations, they also reported arrest of irradiated cells in G_2_ phase. They did not observe any increase in apoptosis but did observe premature senescence that was detected 3 days after irradiation, which is similar to what is observed in fibroblasts and bone marrow mesenchymal stem cells (Serakinci et al., 2007; Tsuboi et al., 2007; Muthna et al., 2010).

In summary, our studies show that despite the induction of p53 and the reduction of PCNA expression within 1 hr after irradiation, there were no significant changes in apoptosis, cell differentiation, self-renewal, and NSCs number. By contrast, our data reveal shifts in the abundance and proliferation of specific multipotential and bipotential progenitors. These findings support the view that SVZ NSCs are radioresistant and reflect their capability to detect and initiate the process of maintaining genomic integrity by slowing progression through the cell cycle, thus providing time for repair of DNA damage. As reviewed above, several studies have shown that the neural precursors of the SGZ of the hippocampus are negatively impacted by irradiation, and the loss of these precursors correlates with memory and cognitive deficits. Therefore, our results do not support our original hypothesis that cranial irradiation adversely affect the NSCs of the SVZ. Rather our data support the alternative hypothesis that head irradiation might cause the dysfunction of progenitors that reside within the cerebral cortices.
